# Identifying and sharing per-and polyfluoroalkyl substances hot-spot areas and exposures in drinking water

**DOI:** 10.1038/s41597-023-02277-x

**Published:** 2023-06-16

**Authors:** Sweta Ojha, P. Travis Thompson, Christian D. Powell, Hunter N. B. Moseley, Kelly G. Pennell

**Affiliations:** 1 University of Kentucky, College of Engineering, Department of Civil Engineering, Lexington, Kentucky USA; 2 University of Kentucky Superfund Research Center (UKSRC), Lexington, Kentucky USA; 3 University of Kentucky, Department of Computer Science (Data Science Program), Lexington, Kentucky USA; 4 University of Kentucky, Department of Molecular and Cellular Biochemistry, Lexington, Kentucky USA

**Keywords:** Environmental impact, Water resources, Sustainability

## Abstract

Exposure to per- and polyfluoroalkyl substances (PFAS) in drinking water is widely recognized as a public health concern. Decision-makers who are responsible for managing PFAS drinking water risks lack the tools to acquire the information they need. In response to this need, we provide a detailed description of a Kentucky dataset that allows decision-makers to visualize potential hot-spot areas and evaluate drinking water systems that may be susceptible to PFAS contamination. The dataset includes information extracted from publicly available sources to create five different maps in ArcGIS Online and highlights potential sources of PFAS contamination in the environment in relation to drinking water systems. As datasets of PFAS drinking water sampling continue to grow as part of evolving regulatory requirements, we used this Kentucky dataset as an example to promote the reuse of this dataset and others like it. We incorporated the FAIR (Findable, Accessible, Interoperable, and Reusable) principles by creating a Figshare item that includes all data and associated metadata with these five ArcGIS maps.

## Background and Summary

Per- and polyfluoroalkyl substances (PFAS) are a large group of thousands of persistent fluorinated synthetic chemicals that have been used since the 1940’s and are widely detected in the environment and humans globally^1–3^. Despite their decades of use, PFAS were not regulated or tracked. On October 18, 2021, the United States Environmental Protection Agency’s (EPA’s) Administrator announced a wide-reaching regulatory approach to addressing PFAS in the environment in the US (e.g. PFAS Strategic Roadmap: EPA’s Commitments to Action 2021–2024). This announcement was significant because it required monitoring, reporting, and development of regulatory standards for PFAS for the first time^4^. This regulatory action spurred states to begin collecting new datasets and assessing exposure pathways, especially datasets related to drinking water sources. These datasets are growing fast and will continue to do so for many years to come.

PFAS contamination sources in the environment have been associated with various PFAS “users” that have used products likely containing PFAS. For example, fire training areas (including airports and military training operations) that use aqueous film-forming foams (AFFF) to extinguish fuel fires, as well as many industrial manufacturing operations are considered common sources of PFAS environmental pollution^5,6^. Numerous studies have demonstrated the detrimental health effects of PFAS^7,8^. Out of the thousands of identified PFAS, perfluorooctanoic acid (PFOA) and perfluorooctane sulfonic acid (PFOS) have been the most studied and have received greatest attention in the media and from regulators. Other PFAS species have been less studied but research on their health effects are emerging and their associated regulatory policies continue evolving^4,7–9^. In July 2022, the National Academy of Sciences, Engineering, and Medicine (NASEM) released Guidance on PFAS Exposure, Testing, and Clinical Follow-Up. This report notes that people live, work, and play in communities where PFAS levels likely exceed standards and there is limited guidance for people to protect themselves. The report summarizes what is known about PFAS toxicity and provides a summary of known effect of PFAS on humans, stating there is sufficient evidence that PFAS exposure is associated with decreased antibody response, dyslipidaemia, decreased infant and fetal growth, and increased risk of kidney cancer. PFAS included in the NASEM evaluation include: perfluorooctanoic acid (PFOA), per-fluorooctanesulfonic acid (PFOS), perfluorohexanesulfonic acid (PFHxS), perfluorononanoic acid (PFNA), perfluorodecanoic acid (PFDA), perflu-oroundecanoic acid (PFuDA), and Methyl-perfluorooctane sulfonamide (MeFOSAA)^10^.

The chemical structure of PFAS is characterized by a hydrophobic alkylated chain with fluorine atoms that are attached to the hydrophilic head^11^. The majority of PFAS do not readily degrade in water and other environmental media. PFAS have been measured in the serum of 95% of Americans; therefore, it is important to determine PFAS sources in the environment and other possible sources of PFAS exposure to humans^12,13^. PFAS have been detected in various environmental media, including drinking water^14^, groundwater^15^, surface water^16^, wastewater treatment plants (WWTP)^17^, soils^18^, and air^19^. PFAS sources in the environment are associated with various sites, and unknown point sources around those sites^20–26^.

There is a high probability of detecting PFAS in public drinking water systems that are close to potential PFAS user sites^27^. A case study was conducted using geospatial and statistical modelling for Kentucky, in which PFAS risk-areas were analysed, identifying potential nearby high-risk drinking water systems. The Ojha *et al*. tool^28^ was verified using the 2019 sampling results generated by the Kentucky Department of Environmental Protection Agency (KDEP). KDEP sampled eighty-one (81) public drinking water plants, of which forty-three (43) use surface water sources and thirty-eight (38) use groundwater sources. KDEP analysed these samples for eight (8) PFAS, including Perfluorobutane sulfonate (PFBS), hexafluoropropylene oxide-dimer (HFDO-DA or Gen-X), perfluoroheptanoic acid (PFHpA), PFHxS, ammonium 4,8-dioxa-3H-perfluorononanoate (ADONA), PFOA, PFOS, and PFNA^29^. The results of the KDEP drinking water sampling confirmed with over 80% accuracy that the Ojha *et al*. tool identified drinking water systems as potential high-risk for PFAS contamination^28^.

In this study, the Ojha *et al*. tool^28^, which was developed using the ArcGIS(Aeronautical Reconnaissance Coverage Geographic Information System) Online mapping and analytics software to perform geospatial analysis and generate maps, is extended to ensure data permanence. The ArcGIS web-based platform does not provide data permanence because users are able to remove their maps from the platform or make them private, which effectively prevents access for other users. Since maps can be deleted, ArcGIS cannot provide permanent unique identifiers like digital object identifiers (DOIs) for user-generated maps.

FAIR (Findable, Accessible, Interoperable and Reusable) is a set of guiding principles for managing publicly available datasets^30,31^. FAIR has been wholeheartedly adopted by the National Institutes of Health under their new Data Management and Sharing Policy that emphasizes making data generated from funded NIH research findable with unique identifiers and search mechanisms, easily accessible, i.e., downloadable, interoperable between software, and reusable for future health-related (meta-)analyses.

For these reasons, we created a Figshare item that includes all data and associated metadata with our five ArcGIS-generated maps. Moreover, the metadata description of the maps in javascript object notation (JSON) format adheres to a proposed draft Minimum Information About Geospatial Information System (MIAGIS) standard we have developed^32^ [A proposed FAIR approach for disseminating geospatial information system maps. Summitted to *Scientific Data*.]. Thus, the (meta)data are saved in the FAIRest way possible given the limitations within ArcGIS, enabling their use by other PFAS stakeholders^33^. A metadata-Toxic Release Inventory (TRI) by the EPA is a useful dataset that is FAIR, and reports discharged chemicals annually.

## Materials and Methods

Five different maps were created in ArcGIS Online that have utility to PFAS researchers and decision makers: Hot-spot map (Map-1), drinking water map (Map-2), Sewer map (Map-3), and SWAPP (Source Water Assessment and Protection Program) map (Map-4). All maps are linked in one comprehensive map (MAP-5) that gives directions to the individual linked maps and interested parties may seek to view and evaluate data from all or any of those data sources. ArcGIS Online is limited in its ability to publish data and cannot be used to create DOIs, which requires datasets to have permanent accessibility. In recognition of this limitation, the authors created an associated public Figshare item with a DOI. All data and associated metadata to create these maps are stored within a downloadable zip archive, which is part of the Figshare item and includes a descriptive draft MIAGIS-compliant JSON metadata file.

Map-1 was created using three different publicly available sources: i) Toxic Release Inventory (TRI) (see Data Records section for more information) from DOD and industries, ii) KDEP-supplied data for hazardous sites associated with state-managed hazardous waste programs, and iii) ourairports.com, a publicly available website that contain all airport locations and coordinates. Indicating scores were assigned to all sites. TRI data from 1987 to present for any state was downloaded from the EPA website at https://www.epa.gov/toxics-release-inventory-tri-program/tri-basic-data-files-calendar-years-1987-present. KDEP-supplied data was request using the form: https://kog.chfs.ky.gov/public/requestaccount/. Airport location data was downloaded from the website: https://ourairports.com/countries/US/.

The data and metadata for Map-2, Map-3 and Map-4 were extracted from the same publicly available source, the Kentucky Geoportal. The KY Geoportal is one of the most used data sources for this research, which provides information about all the water systems of Kentucky. All drinking water lines, sewer lines, all SWAPP zones, wastewater outfalls, and coordinates of drinking water treatment plants were extracted from the KY Geoportal at the following link https://kygeoportal.ky.gov/geoportal/catalog/main/home.page.

### Data sources

#### KY geoportal

The KY Geoportal is one of the most used data sources for this research, which provides information about all the water systems of Kentucky. This platform assists in sharing geospatial data of Kentucky, but geospatial data from all states is not present. All drinking water lines, sewer lines, all SWAPP zones, wastewater outfalls, and coordinates of drinking water treatment plants were extracted from the KY Geoportal and uploaded into ArcGIS Online. The distance between water systems and potential PFAS sources is critical information to know if any water system is potentially at high risk for contamination. The ‘measure’ option is available on the left top-most part of the map and assists in measuring distance between the water systems and potential PFAS sources. The KY Geoportal can be found at the following link- https://kygeoportal.ky.gov/geoportal/catalog/main/home.page. All data and metadata that were used in this research are available online.

#### Toxic release inventory (TRI) data

Companies or industries are required to submit detailed annual reports of toxic pollutants that are manufactured, processed, or eliminated as its waste every year in TRI pursuant to Environmental Protection Agency’s (EPA) regulations since 1988. TRI data from 1987 to present for any state can be downloaded from the EPA website. Industries, types of industries, and coordinates of industries were extracted to make the hot-spot map. https://www.epa.gov/toxics-release-inventory-tri-program/tri-basic-data-files-calendar-years-1987-present is the link where TRI data and metadata can be downloaded.

#### Website for airport locations

The address of all airports that are located throughout the state are extracted from ourairports.com. CSV files downloaded from the site include the airports’ name, longitude, latitude, and type. This website includes information about airports for the whole United States and can be extracted for any state and any time. All data for airports were extracted with longitude and latitude and indicating score are assigned as second highest potential risk. Data for airports for can be extracted from the website: https://ourairports.com/countries/US/.

#### Kentucky department of environmental protection agency (KDEP)

Information about the hazardous waste sites in Kentucky was collected by making a request to KDEP. The request can be done by any individual by either emailing or filling out the request form to KDEP. The agency provides information about the sites managed by the agency and the type of operations conducted at the sites (either it is opened or closed). The csv files provided by KDEP includes the name of sites and their address along with longitude and latitude. Any kind of information from KDEP can be requested by using the form: https://kog.chfs.ky.gov/public/requestaccount/.

## Methods

**Fig. 1:**
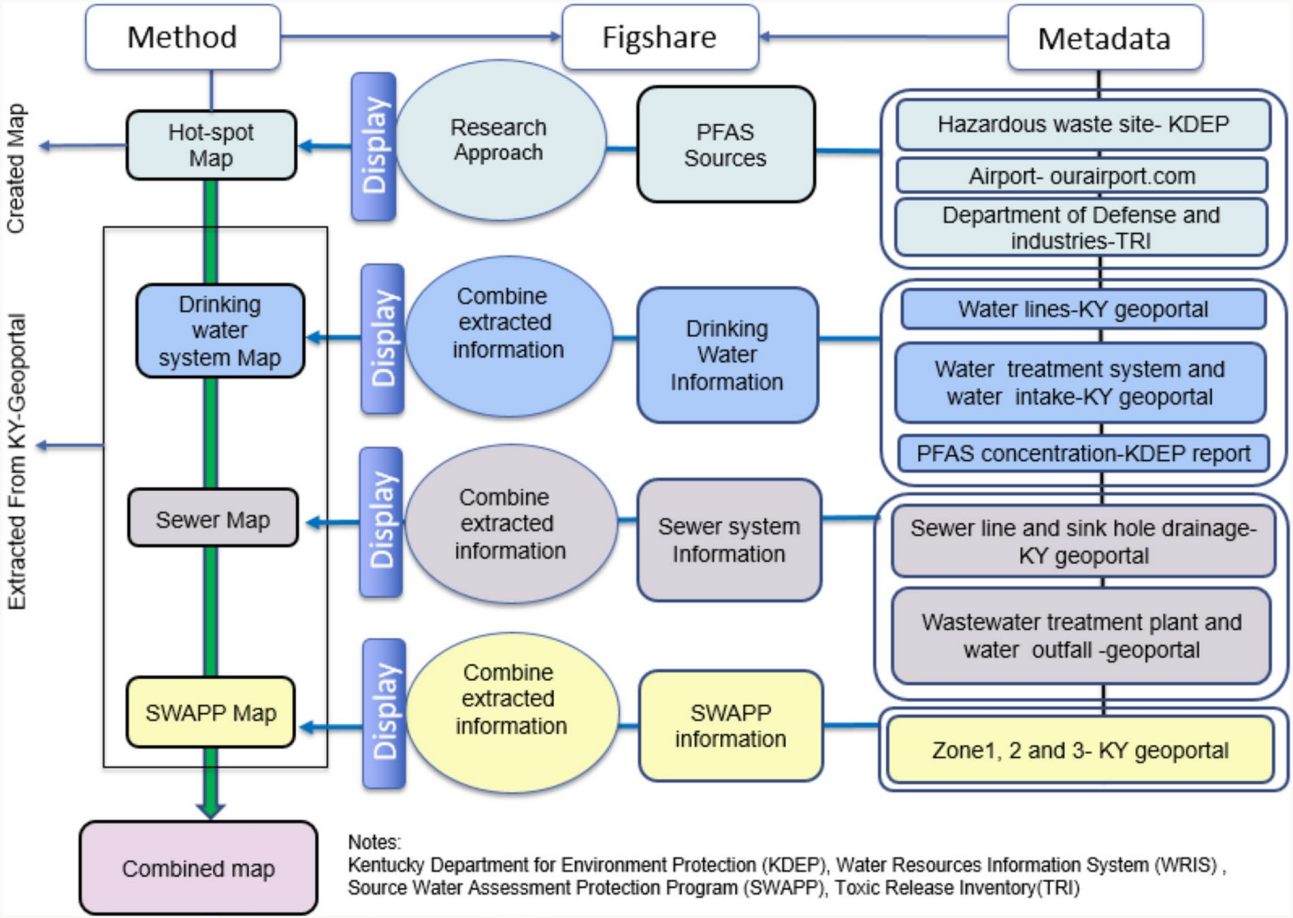
A general overview of the procedures and data sources for creating water maps and a hot-spot map for PFAS.

The methods (M1–4) below include the procedure to make hot-spot maps for PFAS and other linked water systems maps in ArcGIS. A general overview of the map creation and deposition procedures are shown in Fig. 1. We used the draft Minimum Information About Geospatial Information System (MIAGIS) deposition procedures described in Thompson *et al*.^32^ to generate a deposition that was uploaded to Figshare.

### M1. Activation and access of an ArcGIS account

Three different types of ArcGIS Online accounts can be used: an organizational account via a license, a public account using ArcGIS login, or a public account using social login. The process to access or create these accounts are explained below.

#### M1.1. Accessing an organizational account

**Fig. 2:**
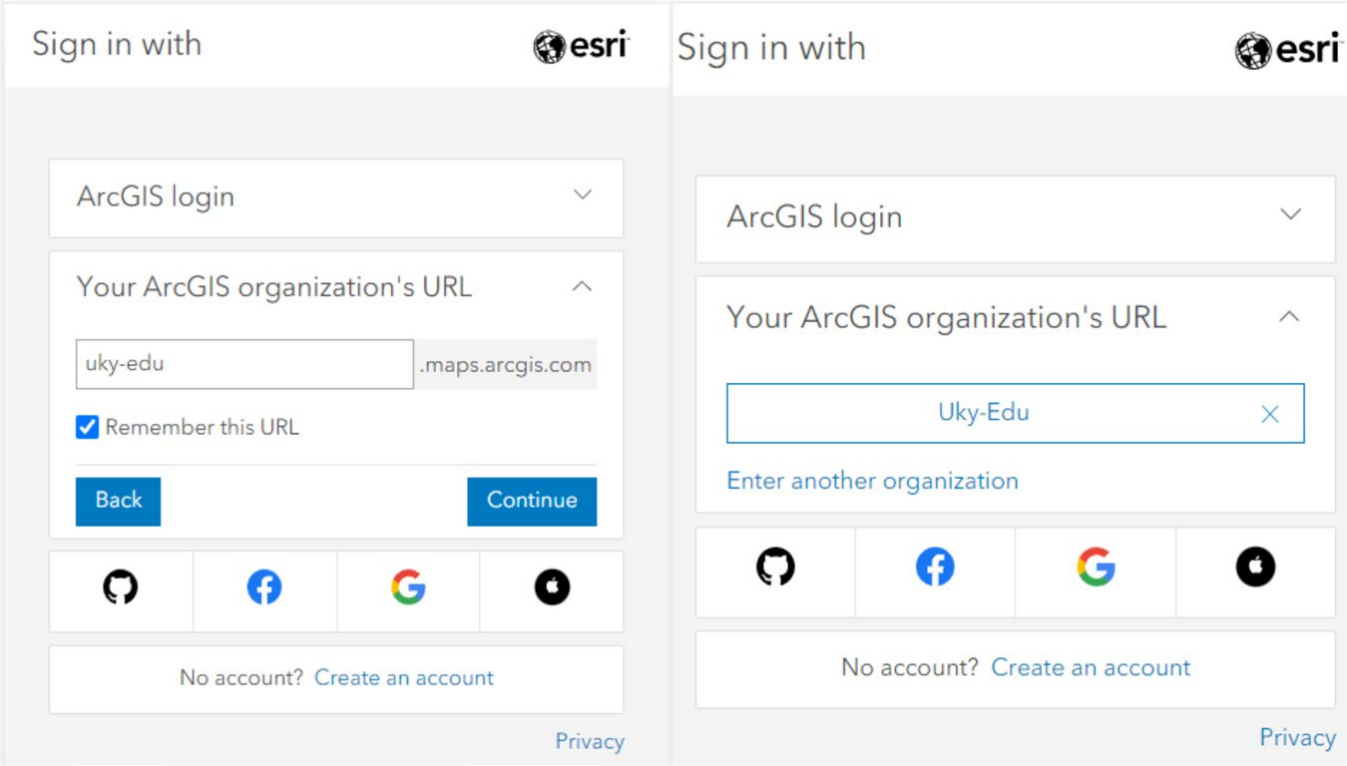
Log-in example of organizational account.
- Click the button with your organization’s URL. Then click the blue button with your organization’s name. This should redirect you to your organization’s login.
- Enter the username and password for your organization.
- The organizational account is created after clicking “**create account**”.

- If you do not have an account through your organization, you should contact the individual managing the organizational account and request access through the organization’s license.
- Use the link https://www.arcgis.com/home/signin.html and **Sign in** directly or select the website (https://www.arcgis.com/home/index.html) and **Sign in**.
- Select and expand “**Your ArcGIS organization’s URL**” (Universal Resource Locater) section from the login screen.
- Enter the organization’s URL, “uky-edu” for example, click the “**Remember this URL**” checkbox, and click “**Continue**”. The screen should look like Fig. 2 below.

##### M1.1.1. Creating a group under an organizational account and adding users.

- Sign into the organization account and verify if the account has permission to invite other organizational members.
- Click “**Organization**” situated at the top of the site. Click “**Invite** **members**” under the “**Members**” tab.
- Click on “**Add members”, “notify them via email**”, and click “**Next**”.
- Select “**New member**”.
- Fill in all required fields (First name, last name, Email address and username).
- Click “**New members from a file**” to upload information about members.
- Create a text or.csv file that contains **Email, First name, Last name, Username, First name, Last name, type, and Role**.

- For example: swetaojha@gmail.com, Sweta, Ojha, Sojha, GIS beginner, student

h. After the member list is reviewed, and the information is verified, click “**Next**”.
i. In the set member properties Panel, licenses can be added by selecting “**add on license**”. Click “**Manage**” in the **Add-on-license** section. Select suitable add-on licenses are for the member and click “**Save**”.
j. From the **Group** section, add the new member to groups in organizations. Click “**Manage**”, select the desired group, and click “**Save**”.
k. Select the “**Settings**” section and modify the following member settings (Profile visibility, language, number, date format and start page of member). Click “**Next**”.
l. Review the summary details. Select “**Compile member list**” to make the change.
m. Select “**Email setting**”. The email message is modified.
n. Click “**Add members**” when everything is finished.

#### M1.2. Creating a public account using an ArcGIS login

- Use the link https://www.arcgis.com/home/signin.html and **Sign in** directly or select the website (https://www.arcgis.com/home/index.html) and **Sign in**.
- Select “**Create an account**”.
- Select “**Create an ArcGIS Public Account**” under **Create a public account**.
- Fill in the required information, i.e., first name, last name and email address typed in their respective places.
- The privacy policy and ArcGIS Online terms of use should be read. Click the box to agree to the terms and policy of ArcGIS Online. Click ‘’**Next**”. A confirmation email will be sent to the email by ArcGIS Online with a link to confirm the email address.
- After clicking the link in the email provide a **Username**. (Username consists of 6–128 alphanumeric characters, special characters like dot (.), underscore (_), hyphen (-), at sign (@) can be used)
- Enter a **Password** (At least eight characters, at least one letter and one number). Retype password to confirm.
- **Select security question**, an answer should be typed and remembered.
- Click “**create account**” and account is created.

#### M1.3. Creating a public account using a social login

- Use the link https://www.arcgis.com/home/signin.html and **Sign in** directly or select the website (https://www.arcgis.com/home/index.html) and **Sign in**.
- Select “**Create an account**”.
- Select “**Create an ArcGIS Public Account”** under **Create a public account**.
- Select the desired social media account to use. **Facebook, Google, GitHub, or Apple**.
- The prompts should be followed and sign in using the selected social account if needed.
- The new ArcGIS Online account is created.

### M2. Creating a base map for both public and organizational account

The layers or maps are generally available on a server and are web-accessible tiles. The tile layer/Basemap can be expanded in the contents panel, but the visibility of sublayers cannot be edited, added, or removed. Suitable background of geographical contents that the user wants to present can be provided by a basemap. The basemap can be selected according to the needs and can be changed through a basemap gallery. A basemap with multiple layers can also be created from a map viewer.

Process to create a basemap:

- **Log in** to created ArcGIS account.
- **Open** Map Viewer Classic and select “**Basemap**” in the toolbar that is in the top-left side of the ArcGIS map.
- The base map panel will appear. **Select** the basemap that is required.
- Information about the basemap can be viewed in the following way:

- Add basemap –at the top of the basemap panel, select current basemap-select option and properties-information about symbol, transparency, blending, and visible range appears.

e. Click “**Save**”, “**Save**”, and click to save new basemap to the map.

### M3. Adding layers for both public and organizational account

Feature layers should be created for every dataset which is intended to be used for visualization. The following steps should be followed to add any other layers on the map.

- From the main menu bar, click “**Map**”.
- From the “My Map” menu bar, click “**Add**”.
- From the **Add** drop down menu, click “**Add Layer from File**”.
- From “Add Layer from File” window, click “**Choose File**”.
- **Select** the file from the computer to make a feature layer from, click “**Open**”.
- Back in the “Add Layer from File” window, click “**IMPORT LAYER**”.
- The **Change Style** menu should appear on the left-hand side of the map. From here, the feature layer can be manipulated to a desired appearance. For details on how to edit feature layers, view this guide: https://learn.arcgis.com/en/projects/create-a-map/.
- Once you have finished modifying the feature layer, return to the **Details** menu on the side of the map and click “**Content**” (this is different from the Content tab in the main menu).
- For the edited feature layer, select “**More Options**”.
- From the “More Options” drop down menu, click “**Save Layer**”.
- In the “Create Item” window, fill out each field then select “**CREATE ITEM**”.
- Repeat steps b through k for each dataset which is intended for visualization.

### M4. Creating maps in ArcGIS for both public and organizational accounts

ArcGIS Online creates an interactive map that helps in visualizing geographic information. When a new account is created, we can see the page with tabs for ArcGIS, Overview, Pricing, Map, Scene, and Group on the top.

The map created in ArcGIS Online can be seen, edited, and analysed as well as be used or seen on mobile devices, desktop applications, and even web browsers. First, sign into your ArcGIS organizational account.

- First, an ArcGIS Online account is created (M1.0. process).
- Suitable basemap and county map is selected.
- The suitable layers are uploaded.
- From the Content bar, click “**+New item**”.’
- Creating Map-1:

i. All sites (without repeating), types of sites and coordinates of all those sites from TRI Data are selected. All airports of the states with their coordinates from ourairports.com are selected. Open request was done in KDEP for all hazardous waste sites, all hazardous waste sites with its coordinates are selected.
ii. All selected data are inserted in a csv file and indicator scores are assigned for every site. (M4.1 Process for indicating score is in data repository).
iii. The created csv file is uploaded into ArcGIS Online and the shown attribute in the Change Style panel is selected to be indicator-score.
iv. The map style is selected to be heat map that analyses risk of the areas with the help of color (Yellow- very risky area, red-risky area, blue – moderate risk area, and white- no risk area).
v. The colors that are applied to density surface are changed and the position of the color ramp slider is adjusted according to the needs of the user.
vi. The optimal visible range is set according to the need of the user. The visible range can be manually set by moving the slider or by clicking Suggest next to Visible Range slider.
vii. The clusters are adjusted for its size, either larger or smaller by adjusting its Area of Influence slider.

f. Creating other maps (Map-2, Map-3, and Map-4)
  i. All required layers for all maps were extracted from the KY Geoportal. The URLs for the layers are in the README file in the Figshare item.

- If feature layers are already created and saved:
  - From the “My Map” menu bar, click “**Add**”.
  - From the Add drop down menu, click “**Search for Layers**” or “**Add Layer from Web**”.
  - Searching for layers allows you to select layers from your content list or other ArcGIS hosted feature layers.
  - Adding a layer from the web can be accomplished by using the URL from the bottom right of each previously created feature layer’s details page.
  ii. The optimal visible range is set according to the need of the user. The visible range can be manually set by moving the slider or by clicking “**Suggest**” next to Visible Range slider.
  iii. Suitable symbols are selected according to the layers.
  iv. The “Add CSV file only” option is easiest when uploading multiple files and feature layers can be created from the uploaded files layer. Click the “**Add CSV file only**” option: 1. Fill out the Title, Tags, and Summary fields, then click Save.
  v. Transparency of the layers can be changed by moving the Transparency slider. The left side is less transparent, and the right side is more transparent.
  vi. From the “My Map” menu, click “**Save**”.
  vii. From the Save dropdown menu, click “**Save as**”.
  viii. In the “Save Map” window, fill out each field then select “**SAVE MAP**”.
  ix. Each feature layer should be added to a single-story map for visualization and should be present in the Content directory of the map Details menu.

#### M4.1. Process to create hot-spot map for both public and organizational accounts

Potential users of PFAS include metal plating facilities, paper manufacturing facilities, textile manufacturing facilities, electronic manufacturing facilities, and facilities that are known to have been contaminated with chemicals (e.g., chlorinated volatile organic compounds (VOCs)) suggestive of site operations with current or past PFAS use. Based on the type of site and historic operations, each of these potential PFAS users were assigned a risk score as previously reported by Guelfo *et al*.^34^. Possible indicating scores are 100, 75, 50 and 25. These scores were also used in a study that performed statistical analysis to prioritize the sampling location for PFAS^28^. On this scale, 100 represents the highest risk, indicating that the facility is highly likely to use or produce PFAS excessively. Sites with its upper magnitude, sources and indicating score are listed in Table 1.

**Table 1:** Potential PFAS users and assigned risk scores.

Risk Score	Facility	Upper Magnitude	Source
100	Department of Defense	10,000 µg/L (28 PFAS)	AFFF*
	Landfill	1,000 µg/L (13 PFAS)	Waste streams from landfills
	Chemical manufacturing Industries	1,000 µg/L (11 PFAS)	PFAS/ Fluoropolymer manufacturer and user
75	Airport	100 µg/L (28 PFAS)	AFFF^*^
	Fire Training Areas	100 µg/L (28 PFAS)	AFFF*
	Petroleum Refineries	10 µg/L (28 PFAS)	AFFF*
50	Textiles	10 µg/L (13 PFAS)	Fluoropolymer coating
	Furniture	10 µg/L (13 PFAS)	Fluoropolymer coating
	Paper	10 µg/L (13 PFAS)	Fluoropolymer coating
25	Rubber/Plastics	10 µg/L (13 PFAS)	Fluoropolymer coating
	Fire Station	Not Available	PFAS Foam
	Fabricated Metal	Not Available	Fluoropolymer coating

*AFFF - Aqueous Film- Forming Foam.

## Data Records

The layer data and metadata for the hot-spot maps, drinking water map, Sewer map, and SWAPP map are available in Figshare via 10.6084/m9.figshare.15218958^35^.

## Technical Validation

**Fig. 3:**
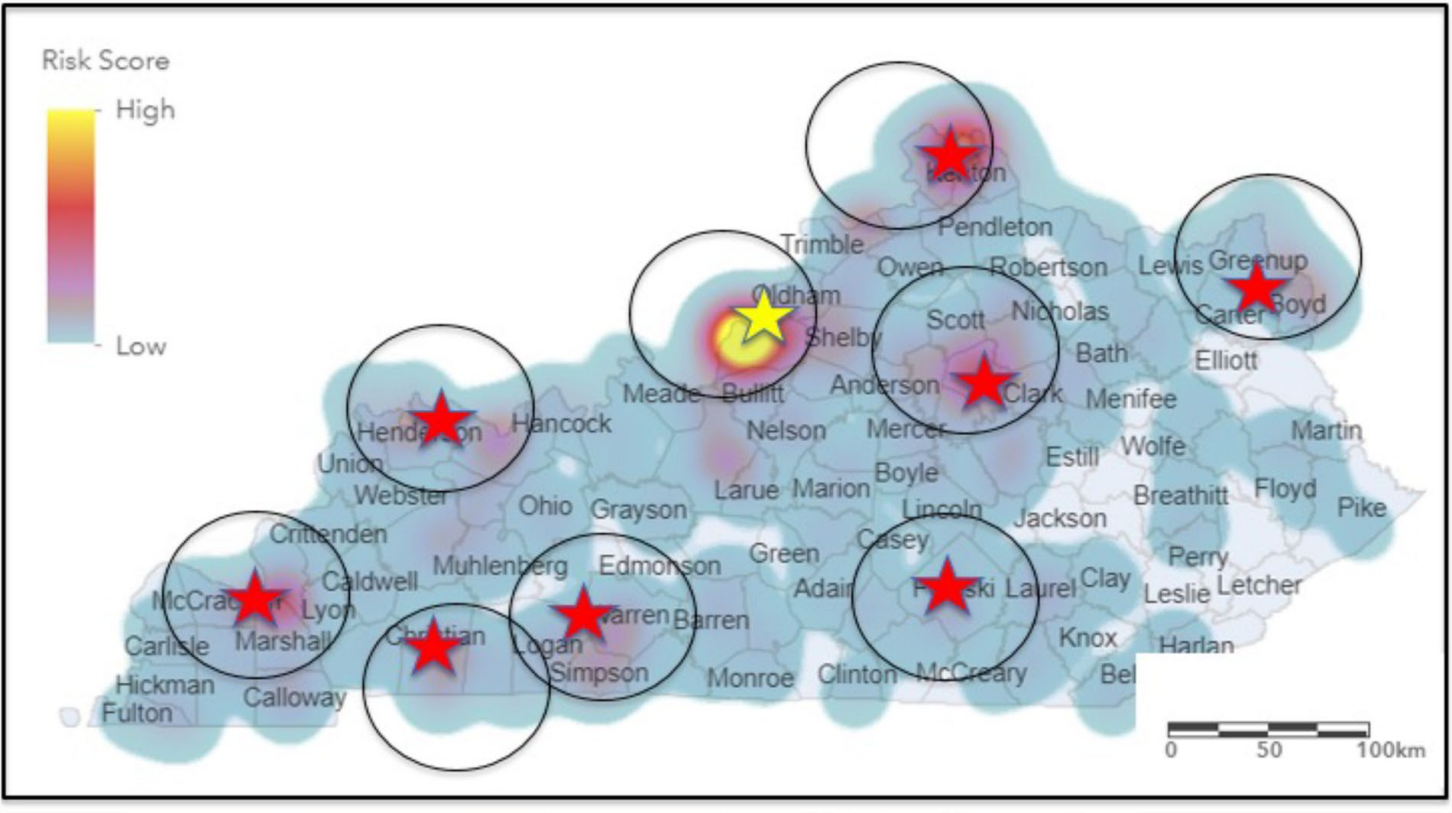
Hot-spot areas in Kentucky showing potential risk areas.

Figure 3 illustrates the hots-pot map, along with some of the potential high-risk areas denoted by yellow and red colors. Table 2 shows the highest PFAS sampling results for drinking water that were published by KDEP in 2019 and 2021^29^. These samples were collected by trained staff from KDEP, and water samples were analysed for eight PFAS. Selected counties, including Henderson, Fayette, Greenup, Christian, Boyd, and McCracken, are denoted in Fig. 3 where KDEP reported detections of high PFAS concentrations.

**Table 2:** Drinking Water Sampling results for 8 PFAS of hot-spot areas in Kentucky by KDEP in 2019 and 2021.

Sampling Year	County	sampling location	Sample type	PFAS concentration (ng/L)								
				PFBS	HFDO-DA	PFHpA	PFHxS	ADONA	PFOA	PFOS	PFNA	Total PFAS
2019	Henderson	Henderson Water-North	DW	ND	12.6	ND	ND	ND	1.43	0	0	14.03
	McCracken	Paducah water works	DW	2.73	4.89	1.12	ND	ND	4.07	4.54	0	17.35
	Greenup	Russell Water company	DW	ND	13.5	1.2	ND	ND	5.62	2.01	0	22.33
	Boyd	Ashland Water works	DW	ND	18.3	ND	ND	ND	4.74	1.96	0	25
	Mason	Maysville Utility Commission	DW	ND	29.7	ND	ND	ND	5.09	1.94	0	36.73
	Greenup	South Shore	DW	8.55	ND	5.02	11	ND	23.2	18.9	0	66.67
2020	Christian	Quarles Spring Branch	DW	13.7	ND	12	135	ND	13.4	249	1.29	424.4
	Christian	Quarles Spring Branch	NP	ND	ND	ND	100	ND	ND	206	ND	306
	Fayette	UT to North Elkhorn Creek	DW	14.3	ND	20.4	14	ND	31.2	6.34	2.4	88.64
	Henderson	Canoe Creek-Downstream	DW	3.83	ND	47.1	1.1	ND	36.6	5.32	29	123
	Jefferson	Duck Spring Branch	DW	6.35	ND	17.6	46	ND	13.8	95.1	2.85	182
	Union	Casey Creek	DW	11.8	ND	5.41	4.8	ND	8.36	21.8	2.56	54.72
	Marshall	Cypress Creek	DW	1.88	ND	ND	2.9	ND	5.45	5.41	37.6	53.25

Note-Not Detected (ND), Drinking Water (DW), NP (Non-Portable), Perfluorobutane sulfonate (PFBS), Hexafluoropropylene oxide-dimer (HFDO-DA or Gen-X), perfluoroheptanoic acid (PFHpA), Perfluorohexane sulfonate (PFHxS), Ammonium 4,8-dioxa-3H-perfluorononanoate (ADONA), PFOA, PFOS, and Perfluorononanoic acid (PFNA). Source: ^28^.

## Usage Notes

All data and associated metadata are stored in JSON format within a downloadable zip archive. The ArcGIS map creation and deposition methodology should be downloaded and carefully read. Any terms or steps that are unfamiliar should be looked up and understood. All steps should be applied in the correct order after downloading the data files from the Figshare item. In the beginning, go slow and make sure every step is executed correctly before moving to the next step^35^.

## Data Availability

The MIAGIS Python package is available on GitHub and the Python Package Index with comprehensive user documentation on GitHub.io: https://github.com/MoseleyBioinformaticsLab/miagis https://pypi.org/project/miagis/ https://moseleybioinformaticslab.github.io/miagis/
